# Reasons given by hypertensive patients for concurrently using traditional and Western medicine at Natalspruit Hospital in the Gauteng Province, South Africa

**DOI:** 10.4102/phcfm.v5i1.458

**Published:** 2013-05-31

**Authors:** Atileombolo A. Lotika, Langalibalele H. Mabuza, Henry I. Okonta

**Affiliations:** 1 Family Medicine and Primary Health Care, Natalspruit Hospital, South Africa; 2 Family Medicine and Primary Health Care, University of Limpopo, South Africa

## Abstract

**Background:**

In 2007, a large number of hypertensive patients seen at Natalspruit Hospital had poor adherent to their anti-hypertension treatment which manifested itself through poor blood pressure control. On enquiry, they revealed that they were also taking traditional medicines.

**Objectives:**

To explore the reasons given by hypertensive patients for concurrently using traditional and Western medicine.

**Methods:**

A qualitative study was conducted amongst nine purposefully selected participants attending treatment at the hospital. Interviews were conducted in the Southern Sotho and IsiZulu languages and were audio-taped. The exploratory question was: ‘Would you tell us why you are taking traditional medicine together with the antihypertensive medicine your are receiving at this hospital?’ The transcribed and translated transcriptions were analysed using the ‘cut and paste’ method to identify themes.

**Results:**

Themes that emerged were that traditional medicine was readily accessible; traditional healers displayed knowledge and confidence in their medicine; traditional medicine was perceived to counteract the side-effects of western medicine; the two streams were perceived to complement each other and both streams could lead to a ‘cure’. Patients were disappointed at the perceived bad attitude of the hospital staff.

**Conclusion:**

The reasons given by hypertensive patients for their concurrent use of traditional and Western medicine centred around patients’ relatively favourable perception of traditional medicine and its practitioners. Western medicine health care practitioners should continue health education on antihypertensive medication in a manner acceptable to patients.

## Abstrait

**Les raisons avancées par les patients souffrant d'hypertension à l'utilisation concomitante de médicaments traditionnels et modernes à l'hôpital de Natalspruit, dans la province sud-africaine du Gauteng**.

**Contexte:**

En 2007, un grand nombre de patients souffrant d'hypertension consultant à l'hôpital de Natalspruit respectaient mal leur traitement contre l'hypertension, ce qui se manifestait par une faible maîtrise de la tension artérielle. Après enquête, ces patients révélaient qu'ils prenaient également des médicaments traditionnels.

**Objectifs:**

Étudier les raisons fournies par les patients souffrant d'hypertension afin de justifier la prise concomitante de médicaments traditionnels et modernes.

**Méthodes:**

Une étude qualitative a été réalisée auprès de neuf participants délibérément choisis fréquentant l'hôpital afin d'y être traités. Les entretiens ont été réalisés en Sesotho et en IsiZulu et enregistrés. La question posée à chacun était la suivante: « Pourriez-vous nous dire pourquoi vous prenez des médicaments traditionnels en même temps que le médicament contre l'hypertension qui vous est prescrit dans cet hôpital? ». Les entretiens retranscrits et traduits ont été analysés à l'aide de la méthode du « copier-coller » afin d'identifier des thèmes.

**Résultats:**

Les thèmes qui ont émergé de cette étude étaient que les médicaments traditionnels étaient aisément accessibles; les guérisseurs se montraient bien informés et confiants en leurs médicaments; les médicaments traditionnels étaient perçus comme compensant les effets secondaires des médicaments modernes; les deux sources de médicaments étaient perçues comme complémentaires et chacune pouvait entrainer une « guérison ». Les patients étaient déçus par l'attitude négative qu'ils percevaient au sein du personnel hospitalier.

**Conclusion:**

Les raisons données par les patients souffrant d'hypertension à l'utilisation concomitante de médicaments traditionnels et modernes tournaient autour de la perception relativement favorable que les patients avaient des médicaments traditionnels et des guérisseurs. Les professionnels de la santé du milieu hospitalier devraient informer les patients sur les médicaments permettant de lutter contre l'hypertension d'une manière acceptable pour ces derniers.

## Introduction

The World Health Organization (WHO) defines traditional medicine (TM) as ‘diverse health practices, approaches, knowledge and beliefs incorporating plant, animal, and/or mineral based medicines, spiritual therapies, manual techniques and exercises applied singularly or in combination to maintain well-being, and to treat, diagnose or prevent illness. TM is a comprehensive term used to refer both to traditional medicine systems such as traditional Chinese medicine, Indian *ayurveda* and Arabic *unani* medicine, and to various forms of indigenous medicine in various countries. Therapies include medication therapies derived from herbs, animal parts and/or minerals, as well as non-medication therapies such as acupuncture, manual therapies and spiritual therapies.’^1^

Oreagba, Oshikoya and Amachree^2^ showed that ‘about 75% to 80% of the world population mainly in developing countries use herbal remedies for primary health care because of their perceived lesser side effects’. Tabassum and Ahmad^3^ state that ‘in the last three decades, research has focused on identification of plants with hypotensive and antihypertensive therapeutic effect’. However, validity of the medicinal value of most plants is still to be determined. Surveys^4, 5, 6^ have shown that between 70% and 80% of all African people seek help from the traditional healers before they even think of Western medicine. A study conducted in Nigeria demonstrated Africans’ belief in traditional medicine and a perception that Western medical practice did not cover all the medical needs of the Nigerian population.7 In Ghana there is an average of one traditional healer for 400 people compared to one conventional doctor for 12 000 people.^8^ In South Africa traditional healers play a significant cultural and spiritual role in the health of the population.^9^ They are consulted first in up to 80% of cases, mainly in the rural and remote areas where there is scarcity of Western medicine.^9^

The principal researcher observed that a large proportion of patients on antihypertensive medication at Natalspruit hospital were taking traditional medicines concurrently, and was prompted to enquire on the reasons for that practice. To our knowledge, there were no published studies that had documented that phenomenon in the Natalspruit area at the time of the study. The aim of this study was to explore and document the reasons given by hypertensive patients at that hospital for their concurrent use of traditional and Western medicine.

## Ethical considerations

Ethical approval for this study was granted by the Research Ethics and Publications Committee (REPC) of the University of Limpopo (Medunsa Campus). Clearance Certificate Number is MP 44/2006.

## Method

### Design and setting

The aim of the study was to explore the reasons given by hypertensive patients on antihypertensive treatment at Natalspruit Hospital, Gauteng Province, who were concurrently using traditional medicine. It was a descriptive, qualitative study using a free attitude interview technique. According to our literature search, there is scanty information on the concurrent use of Western and traditional medicine in the management of hypertension. We chose the qualitative method to explore the subject and gather depth of information as recommended by Hoepfl.^10^

### Sampling and procedure

The study population consisted of all the hypertensive patients seen and managed at Natalspruit hospital who had confirmed their use of traditional medicine. Nine participants who met the inclusion criteria were purposively selected. The inclusion criteria were: 18 years or older, mental stability, willingness and ability to communicate, current resident of Katlehong, and concurrent use of antihypertensive and traditional medicine. Each participant signed the consent form. Recruitment of participants continued until saturation of information was reached at the ninth participant. The languages spoken in Katlehong are Southern Sotho and isiZulu. The exploratory question was ‘Would you tell us why you are taking traditional medicine together with the antihypertensive medicine you are receiving at this hospital?’ Translated into Southern Sotho the question was: *Lebaka ke eng le etsang gore udirise ditlhare tsa setho udi tlhakantse le esta blood pressure*? and into isiZulu: *Kungani usebenzise imithi yesintu, uyihlanganise nalena esikunika yona ekukwelaphenini isifo se high blood pressure?* The interviews were conducted in the participants’ homes by a trained qualitative research associate proficient in the local language. During the interview, only clarification questions were asked on issues raised by a participant – no new questions were asked so as to prevent interference with the participant's line of thought.^11^ To ensure dependability, all participants were asked the same exploratory question. The principal researcher took field notes. The research team had the opportunity to see the traditional and the Western medicine that the participants were using. The interviews were audio-taped and transcribed verbatim by a research associate. Each participant was given feedback on his or her transcription to ensure correctness of captured information, thereby ensuring its trustworthiness. The transcriptions were translated into English by an independent linguistic specialist. The principal researcher read the translated interviews several times to optimise understanding. The emerging themes were identified by using the ‘cut-and-paste’ method.^12^

According to Lincoln and Guba^13^, the trustworthiness of a qualitative study is evaluated by its credibility, transferability, dependability and confirmability. In our study, credibility (the confidence in the truth of the findings) was ensured by prolonged engagement with the participants so as to understand the phenomenon of their concurrent use of traditional and Western medicine in the treatment of hypertension. The interviews were conducted at the participants’ homes to observe them in their own environment so as to gain a deeper understanding of the phenomenon. To ensure transferability, we explain our data collection methodology and the results derived so as to indicate that the findings can be applied in other contexts.^13^ We established dependability (showing that the findings are consistent and could be repeated) by enlisting the services of an independent researcher who was not part of the research team to evaluate and interpret the study results.^13^ Confirmability (the extent to which the study findings are shaped by the respondents and not by the research team)^13^ was ensured by gathering information through the audio-tapes, field notes and confirming the availability of the traditional and Western medicine at the home of each participant (information triangulation).

## Results

Nine participants, one male and eight females, were interviewed. All the participants were Black people and residents of the Katlehong township of Gauteng Province. Their ages ranged between 47 and 73 years, and the majority were unemployed.

### Theme 1: Traditional medicine was accessible

Most of the participants discussed the availability of traditional medicines at their door-step:^14, 15, 16^

‘There were some women who were going house to house selling that and it was in a sealed bottle… I find the stuff readily available’. (P5, Female, 50)

All the participants claimed that they had learnt about the use of traditional medicine to treat their condition from families, friends or neighbours:^17, 18^

‘Just by hearing from someone saying you know I had this to eat and became better … I just said why not use it, I do want to get better.’ (P2, Female, 47)

‘… after hearing that, you also think … um let me also try it maybe it'll make me feel better …’ (P7, Female, 60)

‘I met this young lady selling this stuff, I ask her how it works and she told me how, then I bought it …’ (P4, Male, 64)

### Theme 2: Traditional healers displayed knowledge and confidence

Traditional healers were perceived as confident and readily available educators on their traditional medicine. In one instance, the traditional healer was even willing to leave his contact details for ease of contact by the patient and his next of kin. Traditional healers dedicated time to explain how the patient should take the medication:

‘I had to know, for I told him that once anything goes wrong my children will come for him legally so. He then left his [*contact*] numbers and house number …’ (P8, Female, 58)

And that this potion works [*in a*] certain way, and you drink it after you finish eating [*breakfast*]’. (P2, Female, 47)

In other instances, the traditional healers visited patients at their homes:

‘He [*traditional healer*] also comes [*to your home*] to check how you are doing every month’. (P6, Female, 70)

Participants expressed their satisfaction with traditional healers and their medicine and they perceived their medicine to be effective:

*‘*… after taking it I started feeling much better as compared to before. My condition used to trouble me a lot …’ (P1, Female, 73)

‘ Everybody in the family was impressed and saying you are much stronger. I don't know how to clearly explain it, but it gave me strength and bravery … I was then happy’. (P8, Female, 58)

‘I don't know how to clearly explain it, but it gave me strength and bravery … I was then happy’. (P8, Female, 58)

### Theme 3: Traditional medicine was perceived to counteract Western medicine's side-effects

Most respondents noticed that they started coughing soon after taking their antihypertensive medication. They found that the use of traditional medicine helped to alleviate the cough:

‘There's something that we don't understand concerning these pills [*Western*], once you use them you start coughing and you don't know where this coughing comes from.’ (P5, Female, 50)

‘Yes, they [*traditional medicines*] do [*help with the cough*], and the garlic as well.’ (P3, Female, 68)

‘If I was not coughing so much, I don't think I'll be using herbs all the time’. (P4, Male, 64)

They spread word of the remedy amongst themselves:

‘Well in my experience when you cough, they [*patient colleagues*] will tell you to drink some concoction [*umhlonyane*]’. (P4, Male, 64)

### Theme 4: The two streams were perceived to complement each other

Participants thought that combining traditional and Western medicine yielded better results as the two complemented each other. This made them continue to use both medicine types concurrently:

‘The reason for me is because Western and African medicines complement each other. It helps and heals’. (P9, Female, 48)

‘…when I drink Western medicine with traditional medicine, I get better …’. (P4, Male, 64)

‘…yes, I did [*feel better when taking Western medicine*], but then I did not stop using treatment I got from the hospital.’ (P6, Female, 70)

‘Yes, I think it's okay to drink both the Western and traditional medicine’. (P7, Female, 70)

An opinion was also expressed that using both streams of medicine could actually lead to a ‘cure’ since they had been told that no cure for hypertension had yet been discovered:

'As I told you I am from Swaziland and … high blood is not curable, so one of them advised me to start drinking imbiza’. (P5, Female, 50)

### Theme 5: Disappointment with conventional health care and staff

All participants were disappointed with some aspects of the conventional health service. There was no consistency in the availability of medication. Patients did not think the system was efficiently run:

‘When you come here you are here for your high blood, and thereafter you join another queue for whatever other illness you might be having.’ (P2, Female, 47)

‘She told me that the type of pills that I have to take they don't have them at the clinic’. (P1, Female, 73)

They were not happy with the poor hospital or clinic service and staff attitudes. Doctors and nurses were perceived to be harsh in their dealing with patients:

‘Don't mention them [*complaints*] at the same time, because they'll start getting angry at you, you mention something else they'll show you the line.’ (P8, Female, 58)

‘…and most patients say that the person [*a doctor*] is harsh, that day I got to see it for myself.’ (P2, Female, 47)

‘At times it's because you have to collect your file and you meet some rude nurse … so by the time you get to see the doctor you are already intimidated’. (P5, Female, 50)

Patients’ wishes were also ignored:

‘They will pile me with Panado's and some small one … [*but*] one thing that I would like them to do for me is that they take me for an x-ray’. (P3, Female, 68)

## Discussion

There was a paucity of literature on qualitative studies on the research topic in general and on hypertension and traditional medicine in particular. Most studies on the topic were on HIV and/or AIDS and diabetes mellitus.^19, 20^ In our study, the main reasons for seeking a cure by using traditional medicines amongst hypertensive patients at Natalspruit Hospital were the accessibility of traditional medicines and satisfaction with traditional healers’ patient care. All the participants in this study also expressed disappointment with the conventional health service. Their frustrations centred on the inconsistent availability of Western medication, different queues for different health problems that prolonged patient push-through times at the health care facility, unacceptable staff attitudes, their ignoring patients’ wishes and the unattended side-effects of Western medication.

In our view, most of these factors could have been treated in a manner more acceptable to patients. The factors mentioned above are not a reflection of the failure of Western medicine *per se*, but of the manner in which health care was dispensed by health personnel to the patients. This finding is consistent with the results of a qualitative study in which disappointment with some aspects of the mainstream medical approach was the main reason why patients consulted homeopaths. According to the authors, ‘if mainstream medicine could cure all illnesses and if doctors met all patients’ expectations, there would be no need for patients to look elsewhere.’ This assertion is affirmed by McWhinney^21^ who states that ‘if the Western medical profession is failing to meet a public need, society will find some way of meeting its own needs, if necessary by turning to a group outside the profession.’

**FIGURE 1:**
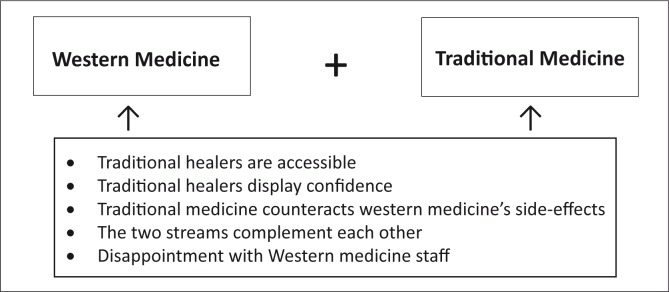
Model of themes: Reasons for the concurrent use of traditional and Western medicine in hypertension management.

### Accessibility of traditional medicine

The disappointed patients in this study turned to traditional medicine as it was readily available on their door-step. Agbor and Naidoo^22^ in Cameroon had a similar finding regarding the accessibility of traditional medicine in their study on the oral care knowledge and practices of traditional healers and why Cameroonians visited traditional healers. In our study, the respondents indicated that they informed one another on the usefulness of traditional medicine. This was comparable to a study by Muhamad, Sharan and Norhasmiria^17^ who found that one of the reasons for patients with breast cancer to seek the advice of traditional healers in Malaysia was a recommendation from a family member or friend. Another study conducted in Lagos State in Nigeria found that up to 78.4% of patients had been influenced by family or friends to use traditional medicine.^20^

A study by Mahonge and Nsenga^23^ in Tanzania found that communities perceived traditional healing as ‘their important, useful and readily available primary health care’. Traditional healers are perceived by patients as culturally acceptable since they explain illness and misfortune in terms familiar to their patients’ local belief systems.^24, 25^ Patients perceive traditional healers to possess a vast store of knowledge locked up in African tradition and unknown to Western medicine.^26^ This vast store of knowledge is culturally transmitted via the symbolic communication by language.^27^ It was no surprise, therefore, that all participants in this study had learnt about the use of traditional medicine through sharing personal experiences with families, friends and neighbours. A study on factors associated with treatment compliance in hypertension in Southwest Nigerian patients reported that patients resorted to traditional medicine and medication from chemists because they could not afford hospital fees and that, unlike Western medicine, they did not have to ‘drink traditional medicine every day’.^28^

#### The complementary systems

Participants perceived that combining traditional and Western medicine yielded better results as the two complemented each other. An opinion was also expressed that using both streams of medicine could actually lead to a ‘cure’ since the patients had been told that no cure for hypertension had yet been discovered. That led to a continuous search for ‘the permanent solution’ in the form of traditional medicine, while also taking the Western medicine. In their qualitative study on why breast cancer patients consulted traditional healers in Malaysia, Muhamad, Sharan and Norhasmiria^17^ also found that the participants perceived traditional medicine as compatible with Western medicine and that using both increased the benefits of both.

The perceived effectiveness of traditional medicine and the satisfaction derived from complementing Western medicine with traditional medicine was noted in this study. Those perceptions were shared with other patients who had been disappointed with Western service provision and this, in turn, led to their easy induction into the concurrent use of traditional medicine. The effectiveness of most traditional medical remedies have not been scientifically studied and proven,^29^ but those who use traditional medicine out of preference or necessity believe in their effectiveness and will probably continue to use them in the foreseeable future.^30^

According to Abdullahi,^16^ traditional medicine is ‘the oldest form of health care system that has stood the test of time’. It gains acceptability because it is practiced within the socio-cultural context of a community, thereby preserving the cultural heritage of the community. Unlike Western medicine, traditional medicine is perceived as holistic towards patient care.^31^ The Australian Aboriginal communities use traditional medicine for the treatment of cancer as a way of reconnecting ‘with their heritage, land, culture and the spirits of their ancestors, bringing peace of mind during their illness.’^32^ In 2008, a study conducted by the World Health Organisation and Health Action International,^33^ revealed that low and middle-income countries were unable to afford Western medicine, leading to a large-scale use of the alternative, accessible traditional medicine by their populations. In other instances, traditional medicine is used as a result of health conditions which have a stigma attached, example sexually transmitted infections, and those perceived to have resulted from supernatural causes, like mental illnesses.^34^ There was no suggestion by the participants in our study that there was stigma attached to hypertension nor that it resulted from supernatural causes. Barimah and Van Teijlingen^31^ have found that Africans who have migrated to western countries continue to use African traditional medicine in their new countries, which indicates that acculturation does not change their practice of and attitude towards traditional medicine. According to Mander, Ntuli, Diederichs and Mavundla^35^, traditional medicine is used by the South African Black population for health problems where Western medicine is deemed inadequate. In our study, the inadequacy was supplemented by the concurrent use of traditional and Western medicine.

#### Side-effects of Western medicine

Most respondents noticed that they started coughing soon after beginning to take their antihypertensive medication. It would seem that the Western health care practitioners did not alert the patients to this side-effect of the medication. Dry cough is a known side-effect of angiotensin converting enzyme inhibitors.^36^ To help alleviate this problem, patients resorted to traditional medicine which they reported to be helping, in keeping with the study conducted by Muhamad, Sharan and Norhasmiria^17^ in which the patients perceived traditional medicine to be helpful in alleviating the side effects of chemotherapy for breast cancer. Patients also reported that they subsequently advised other patients who were not aware of the usefulness of traditional medicines as remedy for the cough. Similar recommendations by family and friends on the use traditional medicine have been reported.^17, 22^

Peltzer and Mngqundaniso^37^ found that a number of HIV positive patients were using traditional medicine and antiretroviral therapy (ART) concurrently in KwaZulu-Natal. They dropped out of ART because of its side effects. One client reported in their study was encouraged to resume ART and she became better but found that the use of traditional medicine helped reduce the side-effects. Although their study was on a different condition than ours, the problem with side effects was similar.

None of the participants reported any adverse effect from the use of traditional medicine. However, that did not imply that traditional medicine was totally safe. In a study on herbal medicine use amongst urban residents in Lagos 20.8% of the patients had experienced adverse effects following the use of traditional medicines, such as ‘skin rashes, vomiting, dizziness, frequent stooling, and abdominal pain’.^20^ This was corroborated by a study in Togo where the traditional healers interviewed reported up to 20 side-effects following traditional medicine administration, the most common of which were diarrhoea, abdominal pains, polyuria, general body weakness and vomiting.^38^ In South Africa, up to 68% of deaths caused by acute poisoning amongst indigenous South Africans were directly linked to traditional medicine toxicity.^39^ We think that in our study patients did not mention the side-effects from traditional medicine because they were not specifically asked the question on side effects of traditional medicine. It would have been interesting to find out what adverse effects they experienced following the use of both types of medicine concurrently.

#### Disappointment with conventional health care and staff

Participants were disappointed with some aspects of the conventional health service. They vented their disenchantments with poor hospital or clinic service and staff attitudes. Doctors were perceived to be harsh in their dealing with patients. Patients’ wishes were also ignored. The study by Muhamad, Sharan and Norhasmiria^17^ found that the delay caused by a long waiting list led patients to seek traditional healers’ help while waiting. In their study, patients reported that they were attracted to traditional healers because of the patient-friendliness of their practices. In our study, traditional healers were perceived to be confident and readily available educators on their traditional medicine. In one instance, a traditional healer was even willing to leave his contact details for ease of contact by the patient and his next of kin. Furthermore, the traditional healers were reported to dedicate time to explain how the patient should take the medication. In other instances, the traditional healers visited patients at their homes. Participants expressed their satisfaction with traditional healers and their medicine and they perceived their medicine to be effective. In Addis Ababa, Ethiopia, Birhan, Giday and Teklehaymanot^24^ found that about two thirds of the patients who visited traditional healers’ clinics did so because they perceived them to be efficacious, while a third visited them because they were dissatisfied with Western medicine practice.

#### The Health Behaviour Model for the phenomenon

The most used health behaviour models are the Health Behavior Models (HBM), the Health Belief Model, Theory of Reasoned Action or Planned Behavior, Social Cognitive Theory and the Trans-theoretical Model.^40^ The HBM suggests that ‘your belief in a personal threat together with your belief in the effectiveness of the proposed behaviour will predict the likelihood of that behaviour’.^41^ In our study, the health-seeking behaviour of the concurrent use of Western and traditional medicine can be explained according to the HBM. The model's four key components are conceptualised as follows: personal vulnerability to the condition, seriousness of the consequences of the condition, effective prevention of the condition through precautionary behaviour and benefits gained through the reduction of the thread.^40^ In keeping with the HBM, we found two factors that mediated the concurrent use practice: the participants’ perception that they were susceptible to side-effects of the Western medicine, hence their use of traditional medicine to off-set those side-effects; and the belief that traditional medicine was effective in controlling their hypertension as it complemented Western medicine. Furthermore, we found that the participants perceived traditional healers to have a holistic approach towards patient care.

#### Limitations of the study

The findings of this study cannot be generalised because of the qualitative study design. However, qualitative studies seek to explore the depth of information in a given phenomenon. The study findings are therefore only transferable to settings with similar environmental and patient characteristics.

## Conclusions and recommendations

The reasons given by hypertensive patients for their concurrent use of traditional and Western medicine at Natalspruit Hospital were that traditional medicine was easily accessible to patients, traditional healers were knowledgeable and confident about their medicine, traditional medicine was able to off-set the side effects of Western medicine, the two streams complemented each other, and patients’ disappointment with the service of conventional health care facility and its staff. Further studies are needed to identify traditional medicine commonly used concurrently with anti-hypertensive medication to address the reasons given by patients in the management of hypertension in the various regions of South Africa so as to investigate the pharmaceutical effect of the concurrent use, and thus facilitate specific patient education on the concurrent use of traditional and Western medicines.
